# Development and Validation of the novel Cuproptosis- and Immune-related Signature for Predicting Prognosis in Hepatocellular Carcinoma

**DOI:** 10.7150/jca.92558

**Published:** 2024-02-25

**Authors:** Yongping Zhang, Ping Sui, Cheng Zhong, Jiansheng Liu

**Affiliations:** 1 Department of Hepatobiliary and Pancreatic Surgery, The First Hospital/First Clinical Medical College of Shanxi Medical University, Taiyuan 030001, Shanxi Province, China.; 2 Department of Oncology, The Affiliated Yantai Yuhuangding Hospital of Qingdao University, Yantai, 264000, Shandong, China.; 3 Department of Orthopedics, The first clinical medical college of Guangzhou University of Chinese Medicine, Guangzhou, 515000, China.; 4 Department of Orthopedics, Jiangmen Hospital of Traditional Chinese Medicine Affiliated to Jinan University, Jiangmen, 52900, China.

**Keywords:** Hepatocellular Carcinoma, Cuproptosis, Immune related genes, Targeted Therapy, qPCR

## Abstract

**Background**: Hepatocellular carcinoma often results in late-stage diagnosis, leading to decreased treatment success. To improve prognosis, this study integrates cuproptosis with immune risk scoring models for HCC patients.

**Method:** We identified differentially expressed genes connected to cuproptosis and immune responses using Pearson correlation. A risk signature was then constructed via LASSO regression, and its robustness was validated in the International Cancer Genome Consortium dataset. Additionally, qPCR confirmed findings in tumor and normal tissues.

**Results**: Eight genes emerged as key prognostic markers from the 110 differentially expressed genes linked to cuproptosis and immunity. A risk-scoring model was developed using gene expression, effectively categorizing patients into low- or high-risk groups. Validated in the ICGC dataset, high-risk patients had significantly reduced survival times. Multivariate Cox regression affirmed the risk signature's independent predictive capability. A clinical nomogram based on the risk signature was generated. Notably, low-risk patients might benefit more from immune checkpoint inhibitors. qPCR and western blotting results substantiated our bioinformatics findings.

**Conclusions**: The genetic risk signature linked to cuproptosis and immunity holds potential as a vital prognostic biomarker for Hepatocellular carcinoma, providing avenues for tailored therapeutic strategies.

## Introduction

Hepatocellular carcinoma (HCC) is an intricate and multistep disease influenced by a combination of genetic and epigenetic factors. It ranks fourth globally in terms of cancer-related mortality rates 1, 2. Hepatitis B, Hepatitis C, excessive alcohol consumption, and genetic predisposition are some of the causes of HCC 3. At present, a clinicopathological staging system is the most important method of prognosticating HCC. It is therefore imperative to develop novel prognostic biomarkers for HCC to predict survival and outline personalized treatment plans.

There are many types of programmed cell death (PCD), including apoptosis, ferroptosis, necroptosis, and cuproptosis, in which the cell dies upon stimulation by an external signal, ranging from physiological to pathological 4-8. This form of programmed cell death (PCD) is induced by copper, called cuproptosis 9. It has been shown that higher serum copper levels are associated with poorer survival in liver cancer 10. According to previous research, liver copper content is closely associated with hepatocellular carcinoma 11, The metabolism of copper affects the microenvironment of tumors as well. Hepatocellular carcinoma (HCC) still lacks a clear understanding of the mechanisms that cause cuproptosis. Thus, it is essential to comprehensively comprehend the association between cuproptosis and HCC and establish novel diagnostic and therapeutic approaches.

In the past few decades, a profound understanding of the intricate interplay between tumors and the immune response has led to notable success in advancing tumor immunotherapy for tumor treatment 12. In spite of this, immunotherapy is only beneficial for a small percentage of the population 13. Tumorigenesis is closely linked to the immune microenvironment as well as to tumor progression and treatment effectiveness 14. There is limited benefit for patients with HCC who take immune checkpoint inhibitors (ICPI) due to the complex liver immune microenvironment and immune suppression mechanisms 15. Due to this, the development of a reliable and comprehensive index that can predict both survival and immune therapy efficacy for HCC patients is urgently needed.

It has been reported that a gene related to copper metabolism may serve as a biomarker for HCC prognosis after reviewing all the evidence presented during this study. HCC patients' survival and response to immunotherapy can also be predicted by immune-related genes in prior research 16, 17. The primary aim of this study is to explore the prognostic implications of the interplay between cuproptosis and immune status in hepatocellular carcinoma (HCC). Specifically, we investigate a novel approach that integrates cuproptosis and immune-related factors to enhance the accuracy of prognosis prediction in HCC. This research aims to address key questions regarding the potential for novel diagnostic and therapeutic strategies in HCC, rooted in the understanding of cuproptosis and immune interactions.

## Materials and Methods

### Data acquisition and preprocessing for Hepatocellular Carcinoma

HCC samples retrieved from the Cancer Genome Atlas (TCGA) database (https://cancergenome.nih.gov/) were subjected to gene expression analysis, along with clinical factors such as survival status, overall survival time, age, gender, and tumor stage. Subsequently, patients lacking overall survival information were excluded from the analyses. The TCGA databases were directly accessed to obtain RNA-seq transcriptome data (FPKM value) and clinicopathological details from 374 HCC tissues and 50 normal liver tissues. Furthermore, an in-depth investigation into genomic mutations of HCC patients, encompassing somatic mutations and copy number variants (CNVs), was conducted. To validate the findings, an additional analysis was performed using 202 normal liver tissues and 243 HCC samples sourced from the ICGC database. Additionally, four samples of HCC and four samples of normal tissue were obtained from the First Hospital of Shanxi Medical University. The ethics Committee of the First Hospital of Shanxi Medical University also approved it. Inclusion criteria are as follows: (1) Patients diagnosed with hepatocellular carcinoma via pathological examination; (2) Patients without severe preoperative cardiac, pulmonary, cerebral, or renal functional disorders. Exclusion criteria are as follows: (1) Pathological examination confirming intrahepatic cholangiocarcinoma or malignancies of other systems; (2) Patients who have received corresponding antitumor treatments prior to surgery.

### Differentially expressed genes associated with cuproptosis and immunity

Through the utilization of the "limma" package, we discerned genes with DEGs between HCC and normal tissues in the TCGA cohorts. The DEGs were identified based on P-values less than 0.05 and |fold change| greater than 2. Additionally, we incorporated data from a previously published article on cuproptosis-related genes (n = 10) and the Immunology Data and Analysis Portal (ImmPort) genes (n = 2483) to form a focused gene set for our investigation. For the identification of cuproptosis-associated DEGs, we executed Pearson correlation analysis between the overall DEGs and cuproptosis-related genes, adhering to the criteria of Correlation Coefficient (Cor) > 0.5 and P-value = 0.05. Similarly, to identify immune-associated DEGs, we employed the same methodology to assess the correlation between the total DEGs and immune hallmark genes. To gain insights into the potential functions of these immune-associated DEGs, we conducted Gene Ontology (GO) enrichment analyses encompassing cellular components (CC), biological processes (BP), and molecular functions (MF). Furthermore, KEGG enrichment analyses were performed to explore their potential functions. To ascertain statistical significance in all our analyses, we set the p-value at 0.05.

### An evaluation of the development and validation of a prognostic risk signature associated with cuproptosis and immune phenotypes

Performing a univariate Cox regression analysis on the overlapping DEGs between cuproptosis and immunity yielded statistically significant results for P-values < 0.05. Subsequently, a LASSO-based Cox regression model was constructed, incorporating genes with prognostic significance from the TCGA training cohort. To determine prognostic coefficients for the genes, lambda values were computed using the "glmnet" package with a 10-fold cross-validation setting. In the ensuing analyses, we identified a risk signature associated with both cuproptosis and immunity, capable of predicting outcomes in Hepatocellular Carcinoma (HCC). The risk score was calculated using the following formula, where Coefi represents the coefficients, and xi represents the expression level of the gene:



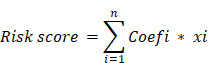



The median risk score of the patients with HCC was then used to categorize them into high- and low-risk groups. Using a log-rank test, we compared the overall survival (OS) time between these groups using Kaplan-Meier analysis. We also performed receiver operating characteristic (ROC) analyses using the survival package, survminer package, and timeROC package to determine the level of predictability for the gene signature based on the survival, survminer, and timeROC packages. Validation of the gene signature's prognostic potential in the ICGC cohort was done using the same formula and statistical methods.

### Evaluation of signature genes' prognostic significance

Each hub gene comprising the prognostic signature was evaluated using univariate Cox regression. Moreover, Kaplan-Meier survival curves for overall survival (OS) were derived using the "survminer" package in order to evaluate the prognostic signature and its relationship to survival outcomes.

### Gene signature associated with prognosis and clinical characteristics

The correlation between the signature genes of individuals and their clinical characteristics was assessed utilizing the "limma" and "ggpubr" packages. Subsequently, two clinical characteristics were identified as significantly associated with the prognosis of HCC patients, employing univariate and multivariate Cox regression analyses, respectively. Based on these clinical characteristics and the risk score, a nomogram was developed to predict the overall survival rates of HCC patients. The construction of the nomograms and the assessment of their predictive ability were performed using the "RMS" package. Calibration curves were plotted to compare the predicted and observed overall survival rates.

### Integrated functional enrichment analyses and gene set enrichment analyses

The statistical significance of the results was determined by analyzing DEGs between high-risk and low-risk groups using the "limma" package. Using the R programming language and the GSVA package, we performed Gene Set Variation Analysis (GSVA) to investigate the biological functions and potential mechanisms associated with the Cuproptosis- and Immune-Associated Risk Signature. Furthermore, Gene Ontology (GO) and Kyoto Encyclopedia of Genes and Genomes (KEGG) pathway analysis was conducted using ClusterProfiler packages. Data visualization was performed using the "ggplot2" package. As part of the preparation for the analysis, a gene set named "c2.cp.kegg.v7.4.symbols" was downloaded directly from the MSigDB repository.

### The prognostic risk score and immune phenotype score (IPS) are related to the tumor microenvironment

By performing single-sample gene set enrichment analysis (ssGSEA) on the RNA-seq expression matrix of Hepatocellular Carcinoma (HCC), the extent of immune cell infiltration within the tumor microenvironment (TME) was evaluated. The effect of immune checkpoint inhibitors (ICIs) was assessed by consulting the cancer immunogenome database (TCIA, https://tcia.at/home). Furthermore, the expression levels of immune checkpoints were compared using boxplots between individuals stratified as high-risk and low-risk.

### Quantitative polymerase chain reaction and RNA isolation

With a NanoDrop 2000 spectrometer (Thermo Fisher, USA), we assessed the purity and concentration of the total RNA isolated from HCC samples using TRIzol reagent (Invitrogen, #15596026). An RT supermix kit (HiScript II Q RT SuperMix, Vazyme, #R222-01) was used to reverse transcription total RNA into cDNA. Supplementary Table 1 and Supplementary Table 2 describe the reaction system and conditions. For all genes except SNHG4, primer sequences were obtained from the PrimerBank database, except for SNHG4, where primer sequences were obtained from a previous study (PMID: 33822671). In Supplementary Table 3, each primer's melting and annealing temperatures were listed (Tm and Tm-5 °C). In Supplementary Table 4 and Supplementary Table 5, the reaction system and conditions for a RT-qPCR assay (ChamQ Universal SYBR RT-qPCR Master Mix, Vazyme, #Q711-02) are described. Standardizing gene expression was done using GAPDH as a reference. Analysis of data was carried out using the 2-ΔCT method. All the primers for RT-qPCR were synthesized by Sangon Biotech company (Guangzhou, China) and the corresponding sequences were listed in Supplementary Table 1.

### Western blotting

To extract protein content from HCC tissue samples, RIPA lysis buffer (Beyotime, #P0013B) supplemented with PMSF protease inhibitor (Beyotime, #ST506, 1:100) was used. SDS-PAGE (Beyotime #P0012A) was used to separate the protein samples, and nitrocellulose membranes (Beyotime, #FFN53) were then used to transfer them. TBST (Beyotime, #ST671) containing 5% skimmed milk was used to block the membranes for 2 hours, followed by overnight incubation with primary antibodies at 4°C. Western blot analysis was performed with the following primary antibodies: KIF18A (abcam, #ab72417, 1:1000), CENPE (proteintech, #28142-1-AP, 1:1000), ATAD5 (abcam, #ab72111, 1:1000), KIAA1841 (Themo Fisher, #PA5-71136, 1:500), CDCA2 (proteintech, #17701-1-AP, 1:1000), PRR11 (abcam, #ab237526, 1:1000), TMEM164 (abcam, #ab122510, 1:250), and GAPDH (proteintech, #10494-1-AP). An additional hour of secondary antibody (abcam, #ab205718; #ab6721) incubation was performed after the primary antibody incubation. Chemiluminescence imaging systems (clinx, #ChemiScope 6000 Touch) were used to detect protein bands using the ECL chromogenic kit (Thermo Fisher, # 32106).

### Statistical analysis

Our statistical analysis was conducted using R software (version 4.1.3). Student's t-tests were used for data that were normally distributed.Wilcoxon tests were used for data that were not normally distributed. Kruskal-Wallis test (one-way ANOVA on ranks) was applied to compare statistically significant differences between multiple groups. The Chi-square test was used to compare categorical variables pairwise. Log-rank tests were used to compare overall survival (OS) times between groups. Two-tailed P-values of at least 0.05 were used to determine statistical significance.

## Results

### Detection and annotation of cuproptosis- and immune-related DEGs in HCC

**Figure 1:**
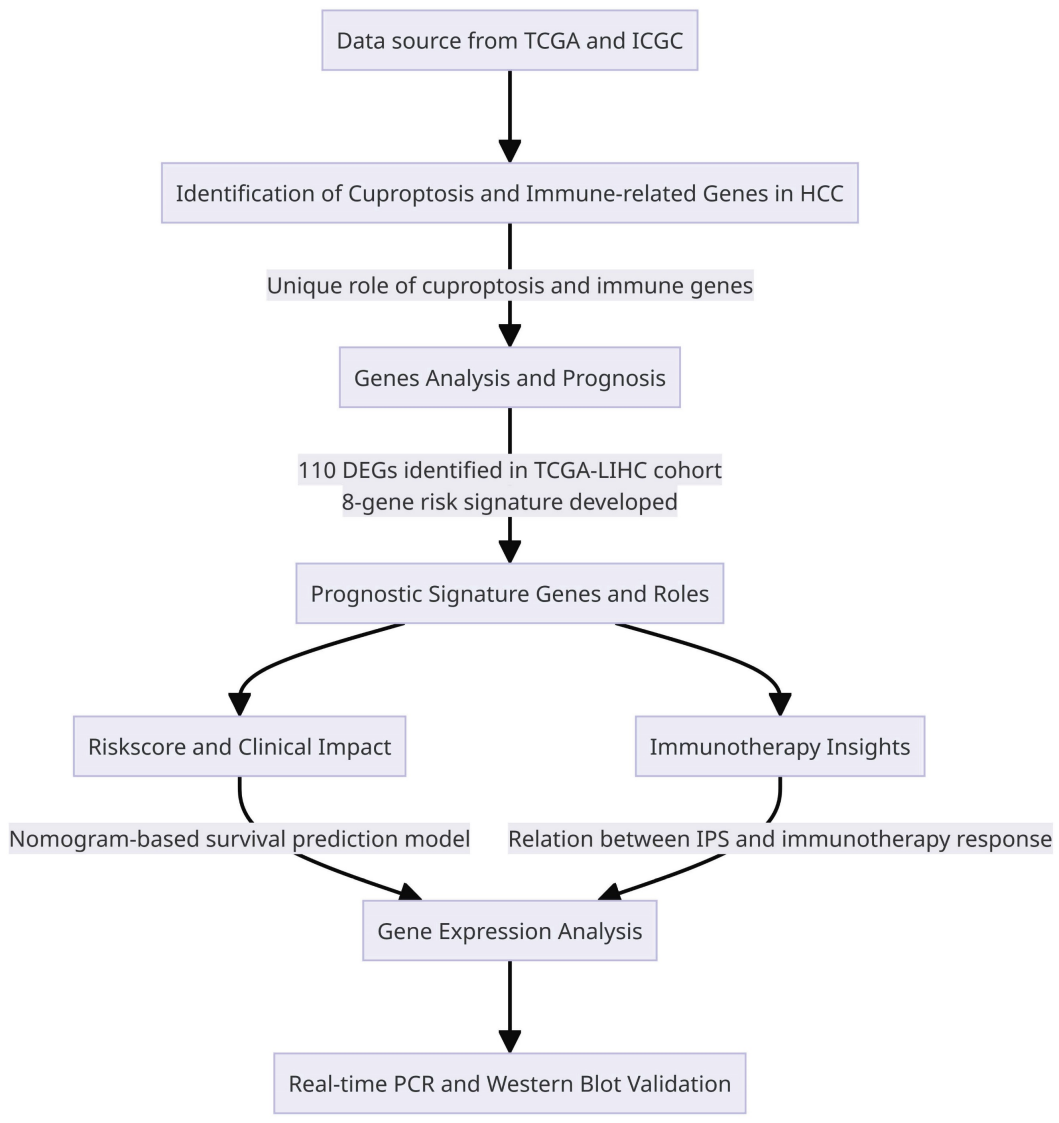
A workflow of the study.

**Figure 2:**
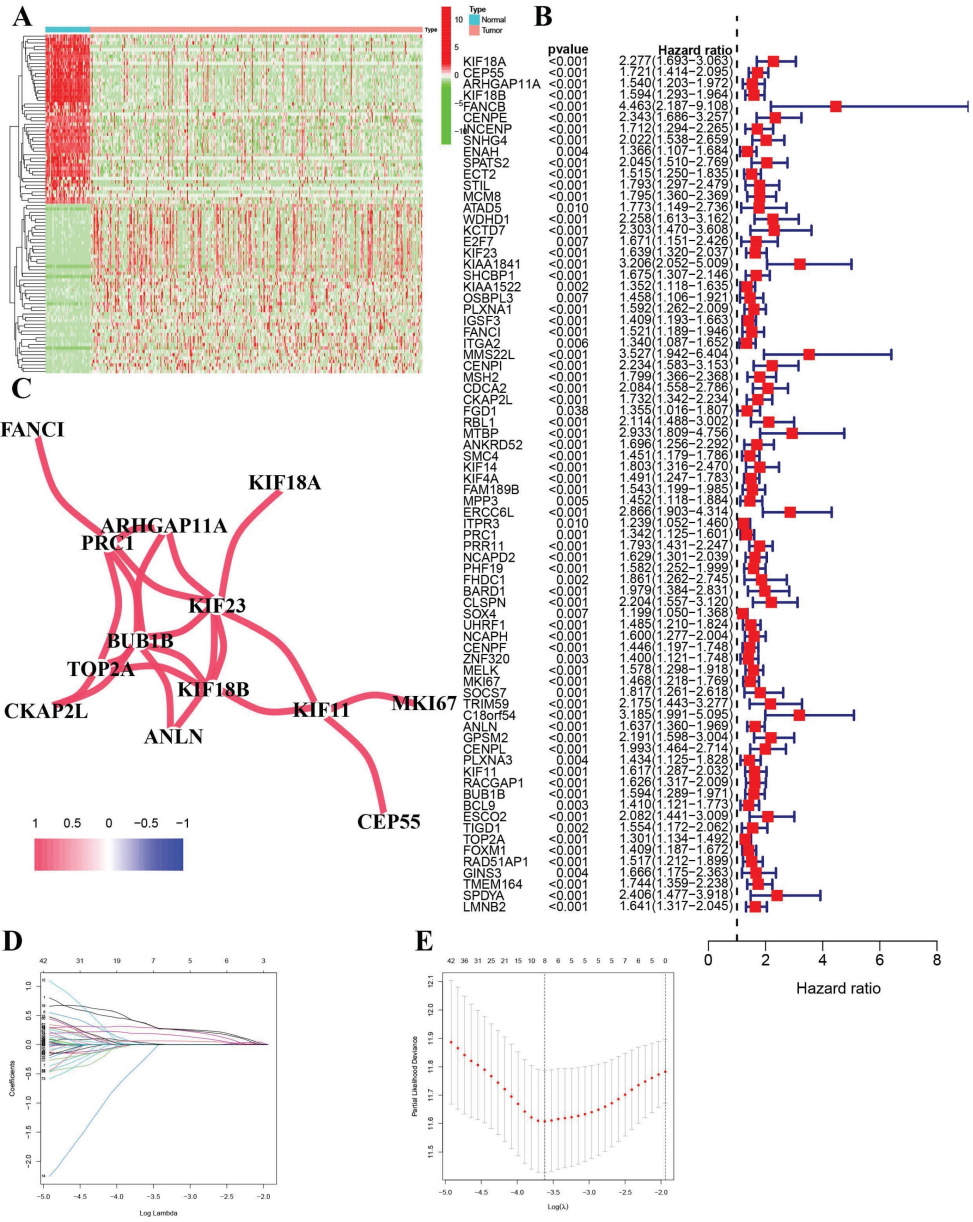
Identification of the prognostic Cuproptosis - and Immune--related DEGs in the TCGA cohort (A) The heatmap showed the expression levels of Cuproptosis - and Immune--related DEGs in tumor and normal tissues, where red indicates high expression and blue indicates low expression. *p < 0.05, **p < 0.01, and ***p < 0.001. (B) Forest plots displaying the outcomes of the univariate Cox regression analysis between the expression of Cuproptosis- and Immune-related DEGs and OS (C) Association network of genes. (D) LASSO parameter profiles of the genes in the training cohort. (E) Parameter profile plot with the log(λ) sequence.

Figure 1 depicts the study workflow. Initially, we utilized the "limma" R package to identify 3917 DEGs from the TCGA-LIHC dataset. Figure 2A was utilized to generate a heatmap, demonstrating the transcriptional levels of these DEGs in both tumor and normal samples. To establish the association between HCC DEGs and cuproptosis-related genes, a Pearson correlation analysis was executed, resulting in the recognition of 111 HCC DEGs linked to cuproptosis. Conducting KEGG pathway analysis and GO enrichment analysis allowed us to gain deeper insights into 2531 DEGs linked to immune-related HCC. The results indicated that cuproptosis-associated DEGs were primarily linked to nuclear division, organelle fission, spindle, cellular senescence, and viral carcinogenesis. Significant correlations were observed between immune-associated DEGs and cell cycle, neuroactive ligand-receptor interactions, and cAMP signaling pathways. Moreover, immune- and cuproptosis-related DEGs were closely linked to cellular function, pathway regulation, and carcinogenesis. (**Supplementary Figure 1A-D**).

### Cuproptosis and immune response in HCC: development and validation of a prognostic signature

A total of 110 DEGs were identified by combining cuproptosis-associated and immune-associated DEGs. Three hundred seventy-four Hepatocellular Carcinoma (HCC) patients from the TCGA database with complete survival information were used in univariate Cox regression analysis to identify genes associated with prognosis. From this analysis, we identified 76 genes that met the criteria with a significance level of P-value less than 0.05 (**Figure 2B**). Furthermore, we calculated correlation coefficients between these genes, revealing predominantly positive regulation among the prognosis-related genes (**Figure 2C**).

In order to determine the most influential weighting coefficients for HCC genes, LASSO Cox regression analyses were conducted. By utilizing the minimal criterion optimal λ value (**Figure 2D, E**), A model of eight gene risk signatures was developed. The risk score was computed as follows: risk score = (0.369 * KIF18A expression) + (2.258 * CENPE expression) + (0.293 * SNHG4 expression) + (-0.240 * ATAD5 expression) + (0.363 * KIAA1841 expression) + (0.032 * CDCA2 expression) + (0.063 * PRR11 expression) + (0.190 * TMEM164 expression).

**Figure 3:**
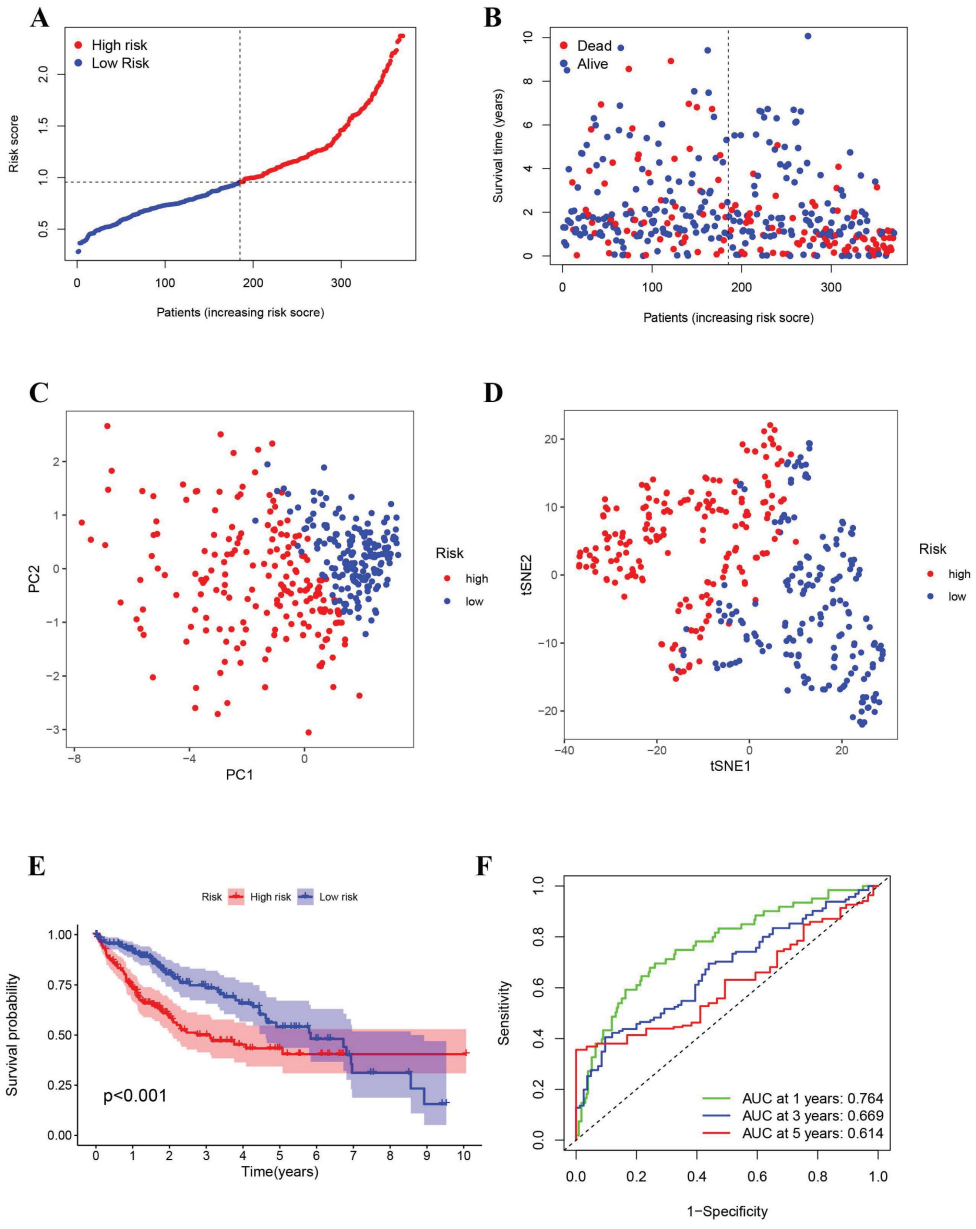
Prognostic analysis of the 8-gene signature in the TCGA cohort (A) Risk score curve shows the distribution of the model and the median score (B) Distribution of survival statuses and risk scores(C) Principal component analysis (PCA) plot (D) t-distributed stochastic neighbor embedding (tSNE) plot (E) Survival analysis in the two risk subgroups. (E) AUC of the risk model (AUC: area under the curve).

After the initial analysis, HCC patients were stratified based on median risk scores into high-risk and low-risk groups (Figure 3A). There was a positive correlation between higher risk scores and higher mortality rates (Figure 3B). Two distinct clusters of patients with divergent risk statuses were identified using Principal Component Analysis (PCA) and Stochastic Neighbor Embedding (t-SNE) (Figure 3C, D). Based on Kaplan-Meier analysis, the overall survival of high-risk groups was significantly shorter than that of low-risk groups, which suggests a better prognosis for low-risk groups (Figure 3E). An analysis of the Receiver Operating Characteristics (ROC) was performed to determine how effective the risk signature was (Figure 3F). ROC curves showed that the risk signature model was accurate at predicting 1-year and 3-year survival according to one-year survival, 0.669 for three-year survival, and 0.614 for five-year survival. The International Cancer Genome Consortium (ICGC) validation dataset showed similar findings. (Supplement Figure 2).

### Prognostic and clinical characteristics validation of eight signature genes risk model

**Figure 4:**
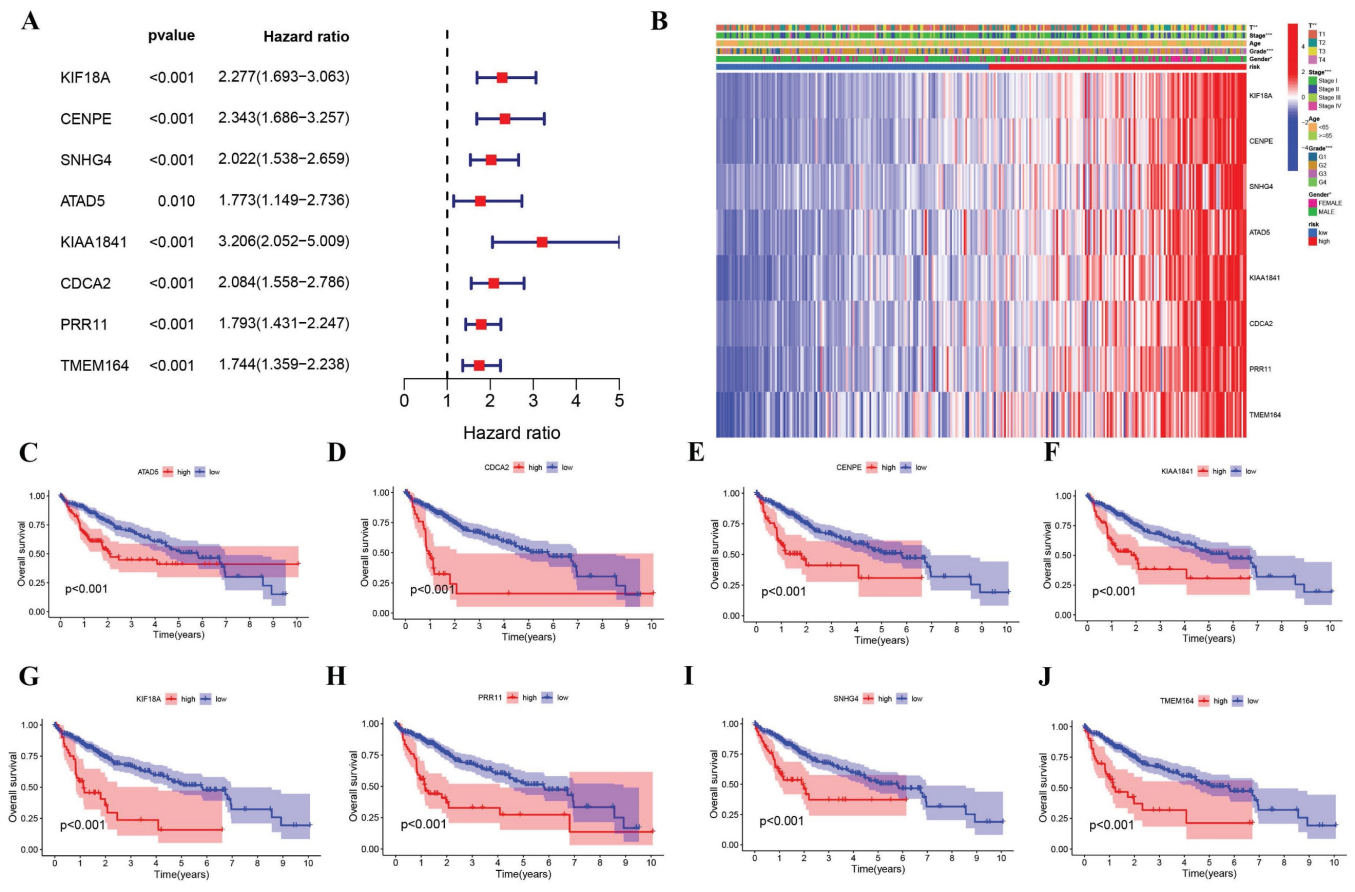
Independent prognostic validation of the eight signature genes. (A) Forest plot of univariate Cox regression analysis based on data from TCGA. (B) Heatmap (blue: low expression.red: high expression) and clinicopathologic characteristics of risk groups (*p < 0.05, **p < 0.01, *** p < 0.001). (C-I) Kaplan-Meier survival of each Cuproptosis - and Immune-related DEGs expression based on data from TCGA.

Based on univariate Cox regression analysis, eight signature genes were identified as being associated with unfavorable outcomes in HCC patients (Figure 4A). As shown in Figure 4B, TCGA participants' clinical and pathological characteristics were correlated with the expression profiles of these eight genes. There was a significant increase in the expression of these genes among the high-risk group, as well as a significant difference in tumor stage, T, grade, and gender. For each of the eight genes, Kaplan-Meier survival curves demonstrated that higher expression levels were associated with worse survival outcomes (Figure 4C-J), correlating with previous findings. The predictive ability of our risk model for HCC was assessed using both univariate and multivariate Cox regression (Supplement Figure 3). Predicting cancer outcomes was found to be possible based on risk scores and tumor stages.

### Developing a nomogram-based survival prediction model

**Figure 5:**
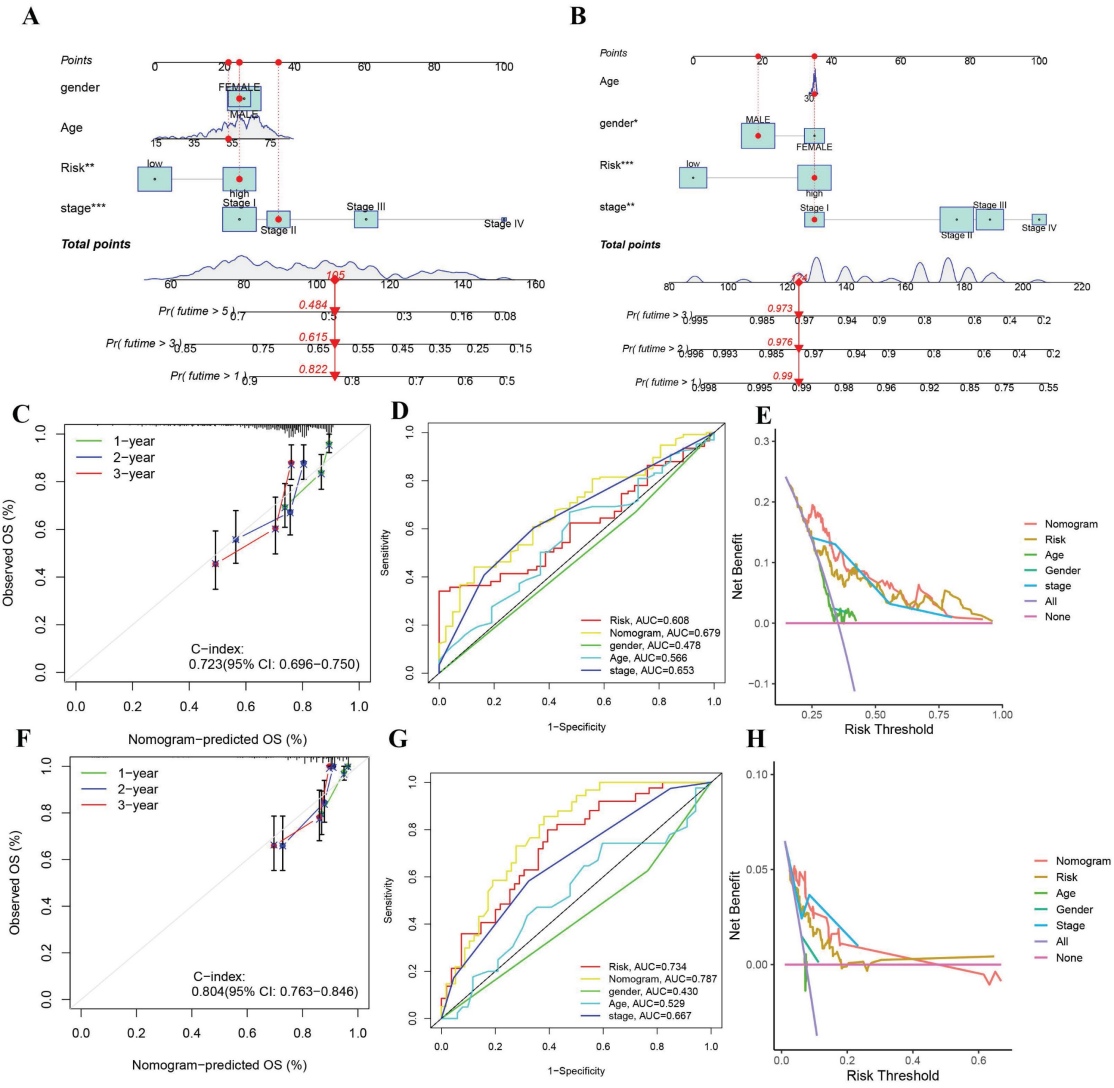
Construction and validation of the riskscore-related nomogram (A-B) Nomogram to predict the 1-year, 3-year, and 5-year overall survival rate of HCC patients in TCGA and ICGC cohorts. (C) Calibration curve for the overall survival nomogram model in the TCGA cohort. (D) ROC curve for the overall survival nomogram model in the TCGA cohort (E) Decision curve analysis of the nomogram in TCGA cohort. (F-H) Calibration curve, ROC curve, DCA curve of the nomogram in ICGC cohort.

The gene risk model's clinical utility in predicting overall survival in HCC patients is limited. Thus, we developed a nomogram that integrates the riskscore with other clinical characteristics to predict overall survival at one, three, and five years. The nomogram plot exhibited the riskscore as a significant predictor of long-term survival (**Figure 5A**). The calibration chart displayed the nomogram's commendable performance in accurately predicting and observing survival rates with high agreement (**Figure 5B**). In the evaluation of prognosis, both univariate and multivariate Cox regression analyses were performed. Independent prognostic factors identified in the univariate Cox regression analysis included the pathological T stage and the nomogram. However, only the nomogram maintained its independence in the prediction through multivariate Cox regression. According to the ROC analysis, the AUC of the nomogram surpassed that of the clinical characteristics, signifying a superior accuracy in forecasting survival compared to the clinical traits. (**Figure 5C**).

### Enrichment analysis of immune- and cuproptosis- associated risk model

**Figure 6:**
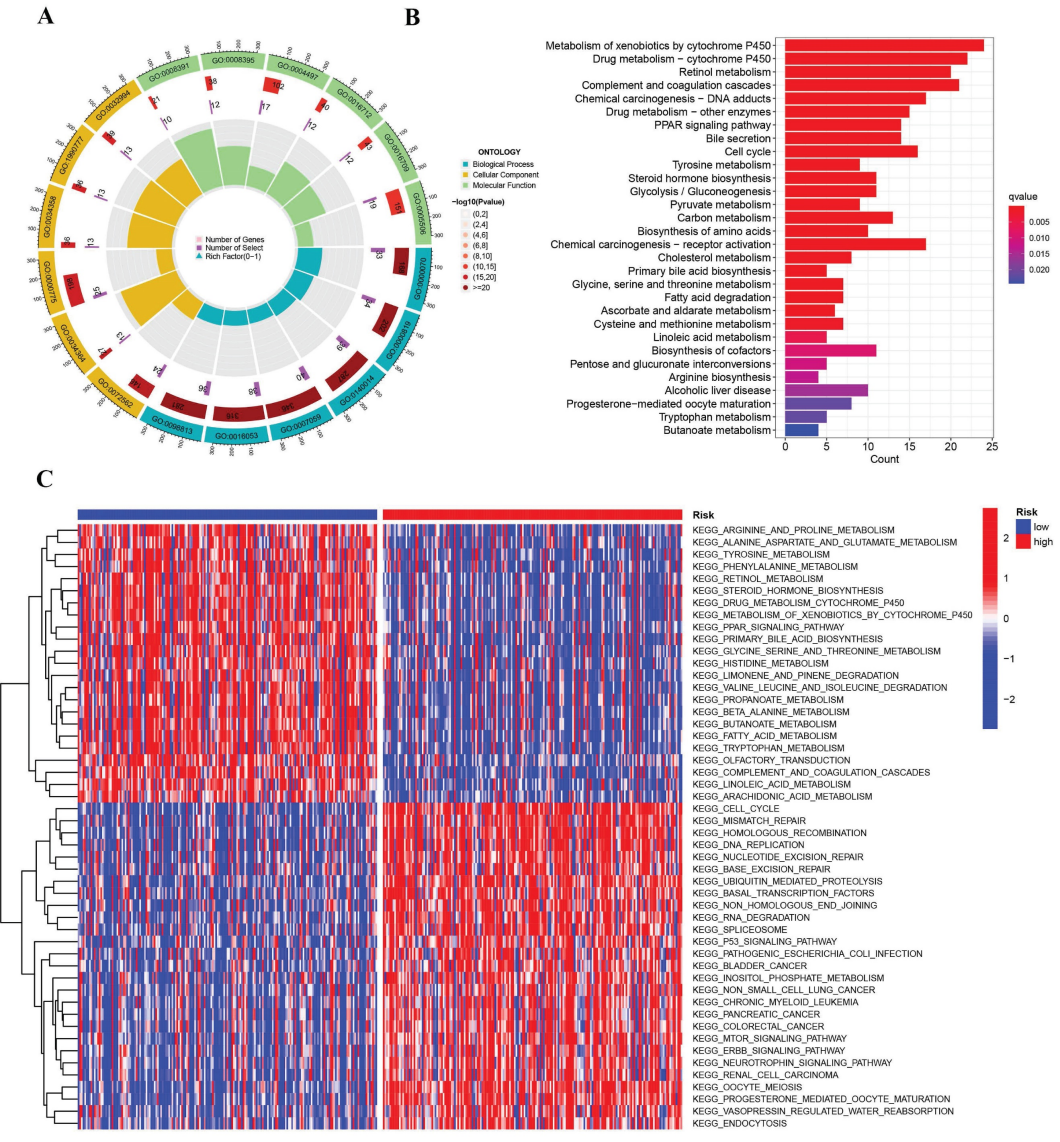
Enrichment analysis of the differentially expressed genes. (A) Barplot graph displayed GO enrichment, with the longer bar represented as the more enriched genes, and the increasing depth of red as the more obvious difference. (B) The results of the KEGG enrichment in TCGA cohort. The abscissa represents the gene ratio, and q-value as the adjusted p-value. (C) GSVA enrichment analysis showed the activation states of biological pathways in high risk group and low risk group. The red represented activated pathways and blue represented inhibited pathways.

In the two risk groups, the gene functions and pathways were scrutinized by recognizing differentially expressed genes (DEGs) having a P-value less than 0.05. Subsequent to this, GO and KEGG pathways were employed to evaluate the DEGs. The DEGs were discerned to be substantially implicated in nuclear division, carboxylic acid biosynthesis, and the segregation of mitotic sister chromatids through GO enrichment analysis (Figure 6A). The analysis of further KEGG pathways uncovered a marked enrichment of DEGs in chemical carcinogenesis DNA adducts, receptor activation linked with chemical carcinogenesis, and the cytochrome P450-mediated metabolism of xenobiotics (Figure 6B). Investigating the two risk groups was also carried out with the application of Gene Set Variation Analysis (GSVA). As revealed in Figure 6C, the evidence suggests that the group at higher risk is characterized by significant enrichments in gene alterations and cellular pathways, encompassing degradation of RNA, the p53 signaling pathway, and the cell cycle.

### The relationship between genomic instability and clinical characteristics based on the risk score model

**Figure 7:**
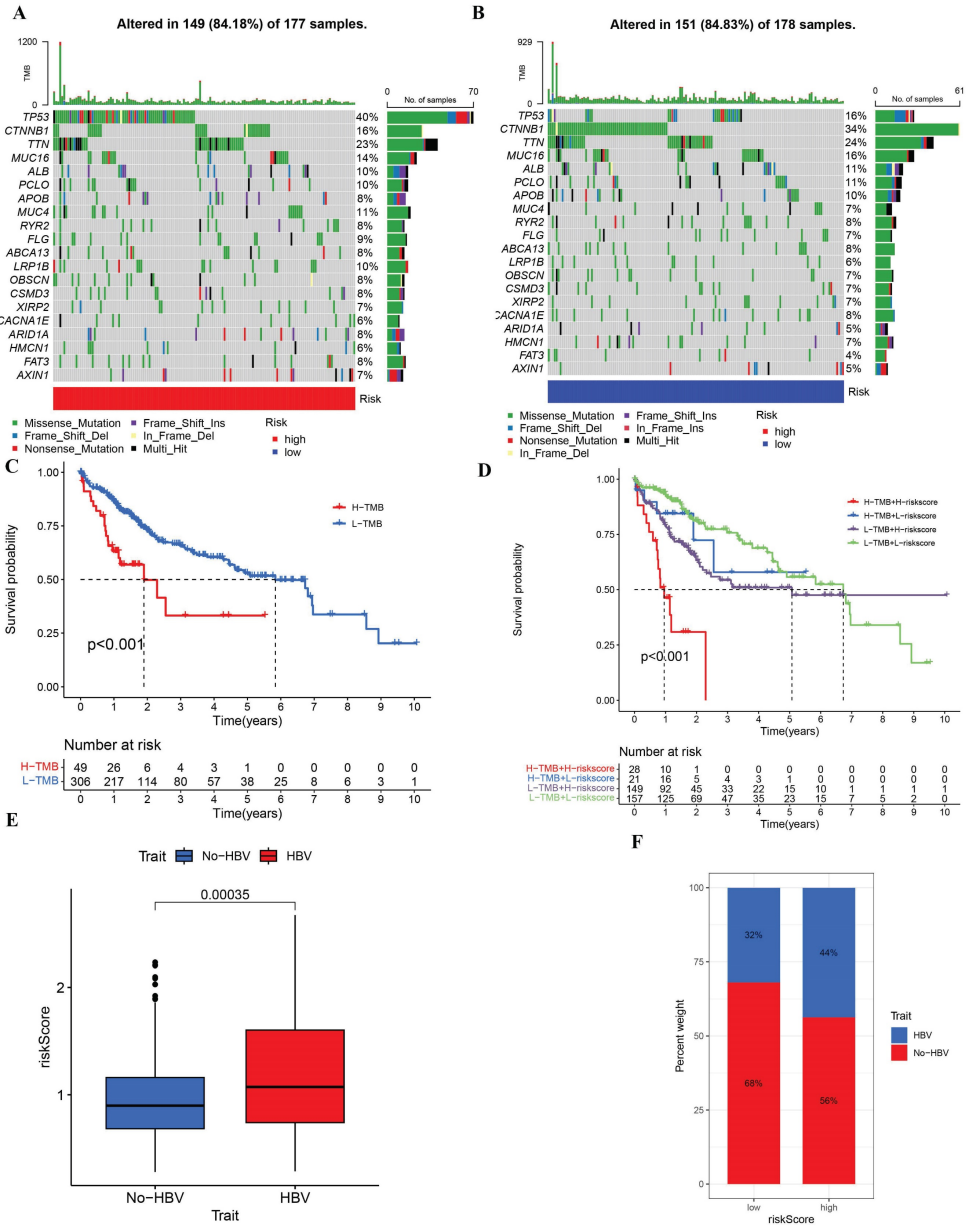
Tumor mutation analysis in TCGA cohort and Differences of riskscore signature in HBV and No-HBV. (A) Waterfall plots in the high-risk group. (B)Waterfall plots in the low-risk group. (C-D) The percent weight of patients with HBV and No-HBV in low or high riskscore group. (E) Survival curves of the high-TMB group and the low-TMB group. (F) Survival curves of the comprehensive analyses between TMB and risk score.

In the prognostic model, tumor somatic mutations were the focus of investigation within this study, employing the TCGA cohort. Differences in mutations between the low-risk and high-risk groups were analyzed through the utilization of the R package maftools. A waterfall chart was constructed to depict the mutation distribution across various risk groups (**Figure 7A, B**), revealing that the most frequent somatic mutations in both high- and low-risk groups were TP53, CTNNB1, and TTN. The high-risk group exhibited a noteworthy difference in the prevalence of TP53 mutations (40%), signifying that TP53 was the gene most often mutated within that group. For the high tumor mutation burden (TMB) and low tumor mutation burden (TMB) subgroups within the risk model, survival analysis was executed (**Figure 7C, D**), demonstrating a deterioration in overall survival rates for the groups with high risk and high TMB, respectively. An association analysis was also performed between the status of HBV infection and the riskscore model, uncovering that the riskscore for patients with HBV was markedly distinct from that for non-HBV patients. Additionally, in the low-risk group, there was an observation of a reduced number of patients who were not infected with HBV. (**Figure 7E, F**).

### The role of risk score in immune infiltration and anti-HCC therapies

The immune characteristics of the signature were verified through the examination of immune cells and risk scores. A majority of immune cells exhibited a positive correlation with risk scores, as depicted in Supplement Figure 4A. A robust association was discerned between higher risk scores and wound healing (immune C1), which manifested as an augmentation in the expression of angiogenic genes and proliferation (Supplement Figure 4B). Additionally, an analysis employing ssGSEA revealed affirmative connections between the activation of type 2 T helper cells, natural killer T cells, dendritic cells, and CD4 T cells (**Supplement Figure 4C**).

**Figure 8:**
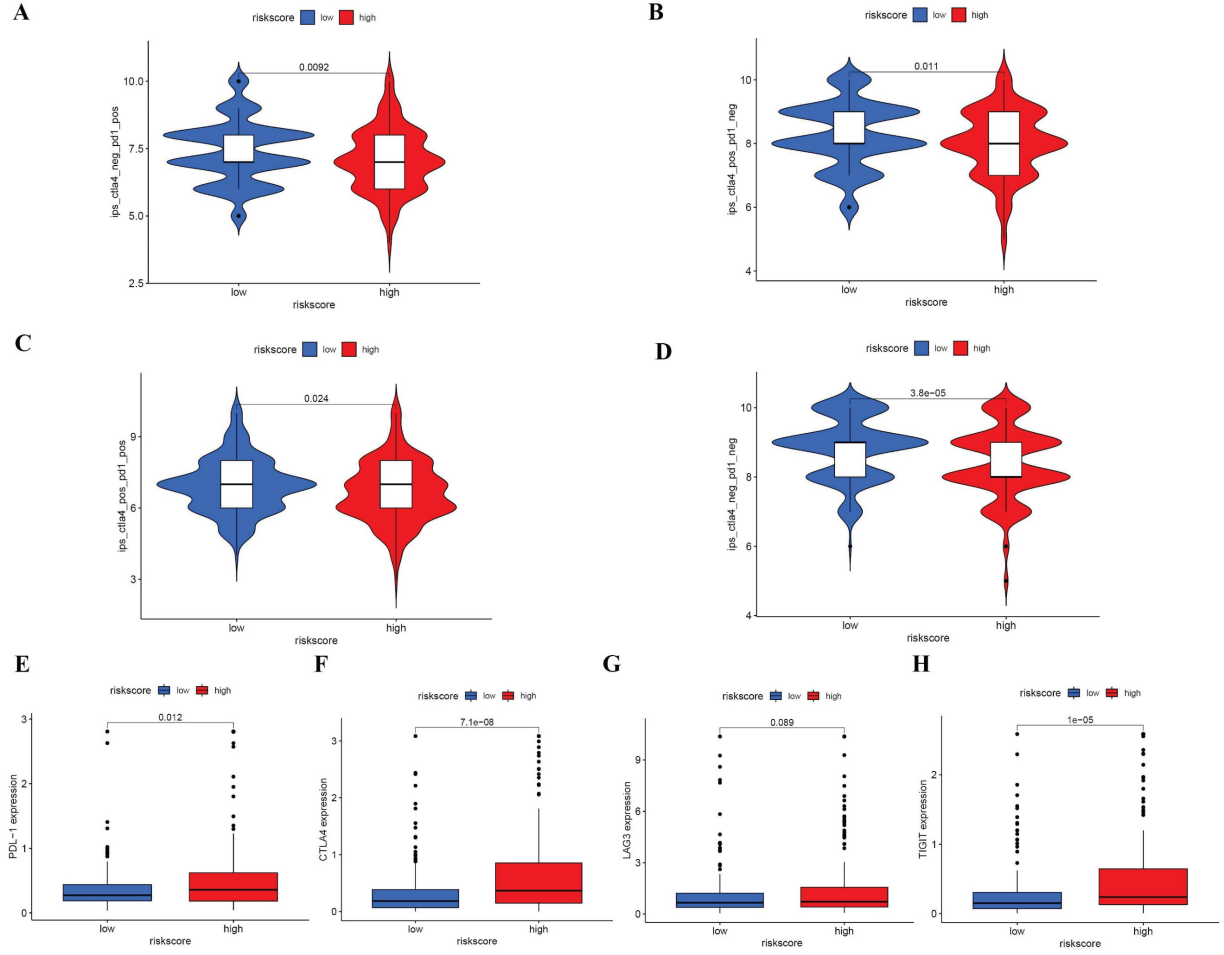
IPS analysis and correlations between risk score and common immune checkpoints (A-D) Comparison of the scores of IPS-CTLA4 blocker, IPS-PD1 blocker, IPS-PD1/CTLA4 blocker and IPS between different risk groups (E) Correlations between risk score and PDL1, CTLA4, LAG3 and TIGIT.

Prior studies have demonstrated that IPS is an effective predictor of immunotherapy response 18, 19. Immune Profile Score (IPS), along with IPS-PD1 blockers, IPS-PD1/CTLA4 blockers, and IPS-CTLA4 blockers, were evaluated and scored. Figure 8A-D indicates that low-risk patients demonstrate a more favorable response to Immune Checkpoint Inhibitors (ICIs) as compared to their high-risk counterparts, inferring that the scores for the low-risk group are elevated. The relationship with the risk score was further explored through the examination of PD1, CTLA-4, LAG-3, and TIGHT. An increased expression of PDL-1, CTLA-4, and TIGHT was observed in the high-risk category (Figure 8E-H). Although no significant variances were found between the groups, an ascending trend in LAG3 was exhibited by high-risk subjects. These observations hint that ICIs may elicit a more favorable response in low-risk patients.

### Validation of risk signature genes model

**Figure 9:**
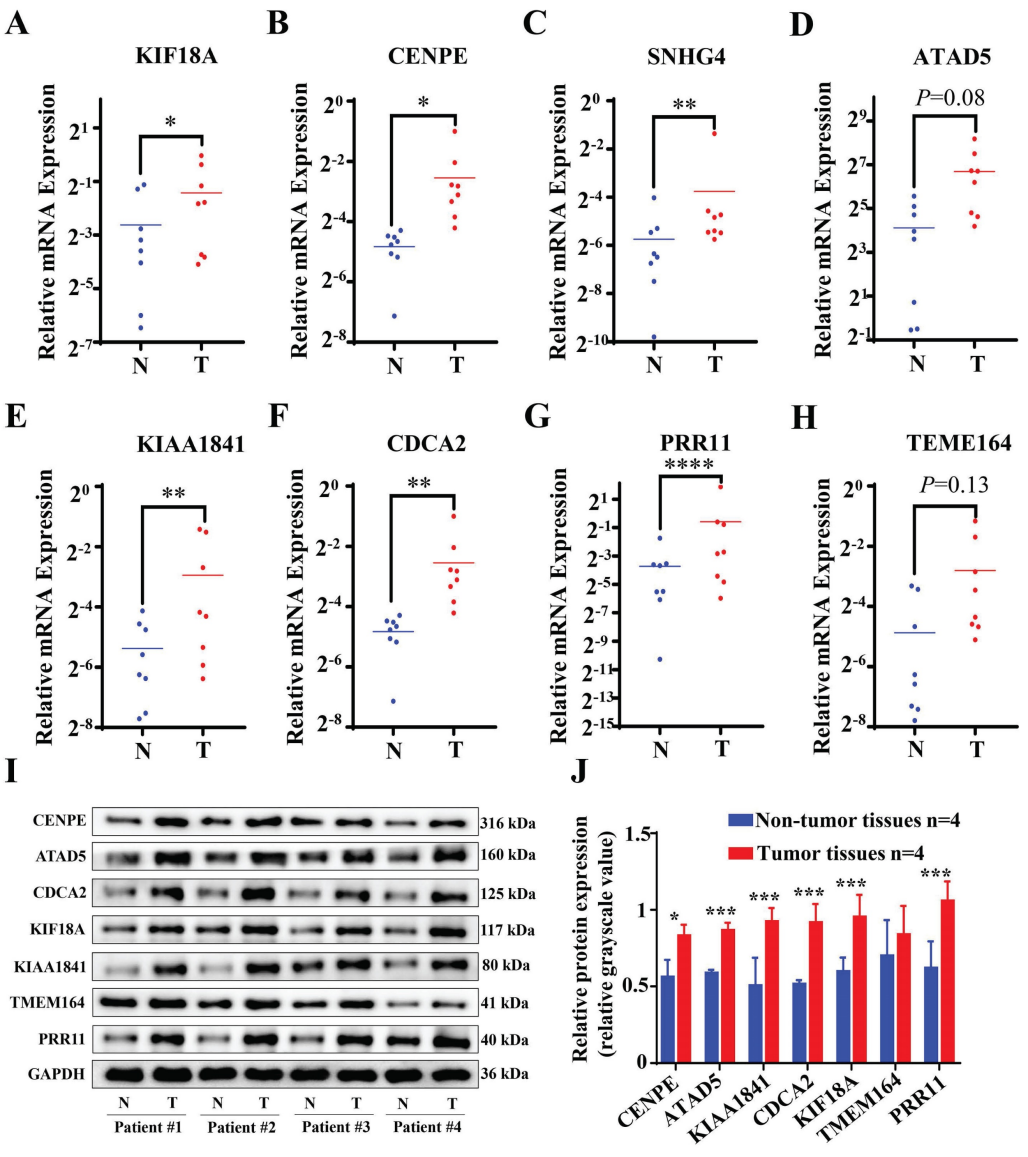
RNA and protein expression levels of Cuproptosis- and Immune-Associated Risk Signature genes in tumor and normal samples. (A-H) qPCR shows expression levels of KIF18A, CENPE, SNHG4, KIAA1841, CDCA2, PRR11, ATAD5 and TEME164. (I-J) Western Blot analysis *p < 0.05, **p < 0.01, ***p < 0.001.

The validation of the results was achieved through a quantitation polymerase chain reaction (qPCR) assessment of genes related to Cuproptosis- and Immune-Associated Risk Signature. An examination of tumor specimens revealed an escalation in the expression of KIF18A, in conjunction with heightened levels of CENPE, SNHG4, KIAA1841, CDCA2, and PRR11 mRNA (**Figure 9A-H**). Although no statistically significant differences were identified between cancer samples and normal samples concerning ATAD5 and TEME164 expressions, a tendency for these genes to be more prominently expressed in cancer specimens was noted. Moreover, Western blot analysis was employed to ascertain the protein concentrations of the model genes, revealing that all the genes were expressed at augmented levels in HCC tissues (**Figure 9I**). These results were consistent with the bioinformatics data. Additionally, we conducted a search on PubMed to identify genes associated with Cuproptosis and obtained a gene signature 20-22.

Subsequently, we gathered models and performed a comparative analysis between our model and others, revealing that our model demonstrated superior accuracy and predictive ability (**Supplement Figure 5**). Employing the IMvigor210 cohort data, our predictive model demonstrates enhanced clinical efficacy over Wang's, especially within the low-risk patient group, evidenced by a statistically significant elevation in the proportion of complete and partial responses (CR/PR) (**Supplement Figure 6**). Furthermore, our model yields more favorable response scores in comparison to Wang's, as denoted by the box plots which reveal a superior distribution of response scores correlating with CR/PR events. Concurrently, in conjunction with data from supplementary Figure 5, the area under the curve (AUC) values within the Receiver Operating Characteristic (ROC) analysis further corroborate the superiority of our model over Wang's signature. This indicates a heightened proficiency of our model in prognosticating positive clinical responses among patients stratified into the low-risk category.

## Discussion

HCC's overall prognosis remains unfavorable despite advances in diagnostic methods and treatment approaches that have contributed to improved outcomes in early-stage patients.^23^. Hence, the identification of a reliable biomarker holds great significance in the assessment of prognosis and treatment outcomes in patients diagnosed with hepatocellular carcinoma (HCC). Recent research findings provide emerging evidence supporting the distinct nature of cuproptosis, which represents a unique form of programmed cell death, separate from other cell death pathways associated with oxidative stress, such as apoptosis, ferroptosis, and necroptosis 24. Numerous studies have demonstrated the significant involvement of copper death-related genes in clear-cell renal cell carcinoma 25 Immune infiltration plays a pivotal role in cancer, and the advancements in the field of immunotherapy have significantly contributed to tumor treatment. Similarly, He *et al.* have made significant contributions in this area. 26 On the basis of immune-related gene expression, they developed a prediction model for treatment outcome.

Within the TCGA-LIHC cohort, this study identified 110 differentially expressed genes (DEGs) pertinent to cuproptosis and immune response, manifesting distinct expression patterns between hepatocellular carcinoma (HCC) and normal liver tissues. To ascertain the prognostic relevance of these DEGs, a univariate Cox regression analysis was employed, leading to the recognition of 76 genes having a connection to the prognosis of HCC. Further, through the application of LASSO Cox regression analysis, an 8-gene risk signature was formulated and subsequently corroborated with an external ICGC dataset. The risk signature, combined with clinical traits, was integrated into a nomogram constructed to forecast the 1-, 2-, and 3-year overall survival (OS) rates in patients diagnosed with HCC.

Using LASSO analysis, a set of eight signature genes was identified (KIF18A, CENPE, SNHG4, ATAD5, KIAA1841, CDCA2, PRR11, and TMEM164). Notably, KIF18A has been implicated in the differentiation and activation of dendritic cells (DCs), suggesting its potential as a therapeutic target for immune-mediated diseases 27. Elevated expression of KIF18A was observed in HCC tissues, stimulating the cell cycle pathway in conjunction with pathways associated with Akt and MMP-7/MMP-9. This led to the promotion of HCC cell proliferation, invasion, and migration 28. Among the spindle checkpoint proteins, human centromere-associated protein (CENPE) induces apoptosis of HCC cells and is antitumor promoting 29. CD4+ T cells are inhibited from undergoing apoptosis when they are exposed to small nucleolar RNA host gene 4 (SNHG4), which is known to be a key component of immunological escape from cancer 30, 31. ATAD5 gene is upregulated by activating E2F1, a key regulator of cell cycle progression, which promotes HBV replication and protects tumor cells from anticancer drugs. B cell division is reduced in the presence of ATAD5 deficiency, which affects Igh recombination 32, 33. KIAA1841 has been identified as being directed to the nucleus, where it is predicted to play a role in regulating transcription, which may function as tumor suppressors in lung tissue. However, there have been limited reports concerning its role in HCC 34. CDCAF2, a protein associated with cell division, contributes to the regulation of protein phosphatase 1 (PP1) g-dependent DNA damage response (DDR) by forming a complex with PP1γ 35. CCND1/CDK4/6 and CCNE1/CDK2 are upregulated by CDCA2 and contribute to HCC proliferation via G1/S transition, according to Wang JH *et al.* 36. Human chromosome 17q22 contains the gene for proline-rich protein 11 (PRR11) 37, which is expressed widely in solid tumors. PRR11 is implicated in the regulation of Wnt expression, contributing to the proliferation and metastasis of HCC cells to some extent 38. TMEM164, classified as a member of the transmembrane protein (TMEM) family, is characterized by its presence in various biological membranes and its independent influence on lung carcinoma prognosis 39. The transmembrane protein 164 (TMEM164) has been shown to be involved in selective autophagosome formation during ferroptosis that is dependent on the ATG5 receptor 40. Furthermore, the functional role of TMEM164 remains poorly understood, with limited available information. A risk score model was formulated based on the expression patterns of the 8 signature genes, and intriguingly, this score displayed associations with cuproptosis, immunity, and prognosis. Favorable outcomes were more commonly observed among low-risk patients compared to those in the high-risk category. The Gene Set Variation Analysis (GSVA) approach was employed to explore the underlying biological processes of these risk groups, revealing that the high-risk category was characterized by dysregulation in areas such as the p53 signaling pathway, DNA replication, RNA degradation, and regulation of the cell cycle. Conversely, the low-risk group exhibited alterations in metabolic pathways, including those related to fatty acids, aspartate, and glutamate. An analysis indicated that HCC patients within the high-risk group manifested a greater prevalence of TP53 mutations and a poorer prognosis, consistent with earlier studies, such as those conducted by Tang et al. The conclusion drawn was that an unfavorable prognosis is likely for patients categorized as high-risk. In addition to the eight-gene risk signature, a nomogram that fused clinical characteristics with the eight-gene risk signature was crafted, serving as a beneficial diagnostic instrument for individualized hepatocellular carcinoma (HCC) assessment.

In the study's ssGSEA expression profile, the low-risk score group was found to be associated with activated CD4 T cells, activated dendritic cells, natural killer T cells, and type 2 T helper cells. Immunotherapy utilizing immune checkpoint inhibitors (ICIs) has been used to treat various advanced malignancies, including hepatocellular carcinoma (HCC). While some cancer patients have responded favorably to this therapy, others have not, emphasizing the necessity to pinpoint the individuals likely to gain from this approach. The investigation sought to understand whether immunity prognostic scores (IPS) correlated with responses to immunotherapy, exploring the link between IPS and risk scores. The findings showed that patients who were scored as low-risk had elevated IPS scores, encompassing IPS-PD1 inhibitors, IPS-PD1/CTLA4 inhibitors, and IPS-CTLA4 inhibitors. This suggested that lower scores might foretell a promising response to immunotherapy. The research also explored the connection between risk scores and immune checkpoints, such as PD1, CTLA-4, LAG-3, and TIGIT. Patients within the high-risk category expressed these markers more than those in the lower risk group, hinting that ICIs might prove more efficacious in lower-risk patients. A risk score model, framed around cuproptosis and immune-related genes, could steer personalized immunotherapy by evaluating immune cell infiltration levels. Real-time PCR was used to analyze the expression of these genes, revealing a significant upregulation of SNHG4, KIF18A, KIAA1841, and CDCA2 within the study. Additionally, ATAD5 and TEME164 were more frequently found in HCC tissues in contrast to normal samples. These findings were further substantiated by Western Blot analysis.

Nonetheless, certain constraints are present in this research. The bioinformatics examinations were grounded on data that is publicly accessible, thus necessitating the formation of more expansive prospective research and additional in vivo and in vitro trials to authenticate these discoveries. Furthermore, a post-translational scrutiny of the genes engaged in cuproptosis and immunity was not performed. Plus, one must not disregard the heterogeneity inherent within tumors. Specific cell types that express particular genes can be identified through transcriptome technology, thereby furnishing an augmented comprehension of the hepatocellular carcinoma (HCC) microenvironment.

## Conclusion

This study presents a newly developed prognostic model incorporating eight genes associated with cuproptosis and immune response, which was achieved through extensive analyses. Moreover, the predictive worth of this model was authenticated using an external ICGC database, offering additional affirmation of its dependability. Notably, our model exhibited the capacity to predict the response to immunotherapy, suggesting its potential as an independent biomarker and therapeutic target in clinical applications.

## Supplementary Material

Supplementary figure legends and tables.

Supplementary figure files.

## Abbreviations

AUC: Area under the curve
DEGs: Differentially expressed genes
GO: Gene Ontology
GSEA: Gene set enrichment analysis
ICGC: International Cancer Genome Consortium
ICIs: Immune checkpoint inhibitors
KEGG: Kyoto Encyclopedia of Genes and Genomes
LASSO: Least absolute shrinkage and selection operator
OS: Overall survival
HCC: Hepatocellular Carcinoma
ROC: Receiver operating characteristic
ssGSEA: Single-sample gene set enrichment analysis
TCGA: The Cancer Genome Atlas
