# Supplementary material for: Development and Validation of the novel Cuproptosis- and Immune-related Signature for Predicting Prognosis in Hepatocellular Carcinoma
Source: J Cancer. 2024 Feb 25;15(8):2260–75. doi: 10.7150/jca.92558 (PMC10937287; doi:10.7150/jca.92558)
Supplement: Supplementary file 1 — Supplementary figure legends and tables. [file jcav15p2260s1.pdf]

Supplementary Figure S1 Functional enrichment analysis of the immune- and cuproptosis-related differentially expressed genes.(A) cuproptosis -associated DEGs GO enrichment results. “BP” stands for “biological process”, “CC” stands for “cellular component” and “MF” stands for “molecular function”. The abscissa represents the gene ratio. (B) cuproptosis -associated DEGs KEGG enrichment results. (C) immune-associated DEGs GO enrichment results. (D) immune-associated DEGs KEGG enrichment results.

Supplementary Figure S2 | Prognostic analysis of the 8-gene signature in the ICGC cohort (A) Risk score curve shows the distribution of the model and the median score (B) Distribution of survival statuses and risk scores(C) Principal component analysis (PCA) plot (D) t-distributed stochastic neighbor embedding (tSNE) plot (E) Survival analysis in the two risk subgroups. (E) AUC of the risk model (AUC: area under the curve).

Supplementary Figure S3 | Univariate and multivariate Cox regression analyses of the riskscore model and Functional enrichment analysis and GSEA based on the risk score model (A-B) Forest plots of univariate and multivariate Cox regression analyses of prognostic signatures and clinical features in the TCGA. (C-D) Forest plots of univariate and multivariate Cox regression analyses of prognostic signatures and clinical features in the ICGC (E, F) GSEA analysis for DEGs between the two groups.

Supplementary Figure S4 | The correlation between the immunity and risk signature (A) Correlations between infiltration of immune cell levels and risk scores. The color blue implies a negative association, the color red denotes a positive association. (B) Risk scores in relation to immune cell subtypes. (C) Infiltration of immune cells between the two group. $*P < 0.05$, $**P < 0.01$, $***P < 0.001$.

Supplementary Figure S5 | Model comparison (A) ROC curve of this article (B-C) ROC curve of other cuproptosis related models. (E-G) Survival analysis of other cuproptosis related models’high- and low risk groups

Supplementary Figure S6 | Comparative Survival and Response Analysis Using the IMvigor210 Cohort for Novel and Wang's Signature (A) Survival curves of our novel signature delineate 'High' and 'Low' risk groups, with a marked survival advantage in the latter ($p = 0.0019$) (B) Response score distribution for our signature demonstrates a significant skew towards better outcomes in CR/PR categories. (C) Proportion of CR/PR versus PD/SD in our signature's risk groups, with 'Low' risk patients showing a higher success rate. (D) Survival curves for Wang's signature, indicating a significant but less stark

survival difference between risk groups (p = 0.0094). (E) Wang's signature response scores, with a narrower difference between treatment outcomes. (F) Treatment response proportions for Wang's signature, depicting less differentiation in CR/PR success between risk groups.

Supplementary Table S1 | Reaction system of reverse transcription

Supplementary Table S2 | Reaction condition of reverse transcription

Supplementary Table S3 | Primer information of the related genes

Supplementary Table S4 | Reaction system of qPCR

Supplementary Table S5 | Reaction condition of qPCR

Supplementary Table S6 | The clinicopathological characteristics of 374 HCC patients in the TCGA cohort

Supplementary Table S7 | The clinicopathological characteristics of 243 HCC patients in the ICGC cohort

Table 1 Reaction system of reverse transcription

component	volume
5×Hiscript II qRT SuperMix	4 μL
total RNA	1 μg
RNase-free ddH_2O	to 20μL

Table 2 Reaction condition of reverse transcription

temperature	time
50 ℃	15 min
85 ℃	5 sec

Table 3 Primer information of the related genes

Primer	Sequence	Melting temperature (Tm)	Annealing temperature (Tm-5℃)
KIF18A-F	TGCTGGGAAGACCCACACTAT	62.6 ℃	57.6
KIF18A-R	GCTGGTGTAAAGTAAGTCCATGA	60 ℃	55 ℃
CENPE-F	GATTCTGCCATACAAGGCTACAA	60.4 ℃	55.4 ℃
CENPE-R	TGCCCTGGGTATAACTCCCAA	62.4 ℃	57.4 ℃
SNHG4-F	GCAGGTGACAGTCTGCATGT	60.6 ℃	55.6 ℃
SNHG4-R	TTTTAAGTCCCCTACCCCCATC	59.15 ℃	54.15 ℃
ATAD5-F	GTGAAGGACTGCGAGATTGAG	60.4 ℃	55.4 ℃
ATAD5-R	TGTCTCTAGTCTTCCCTAGTGGT	61 ℃	56 ℃
KIAA1841-F	GGCTCGTGCCTGGATTAACA	62.2 ℃	57.2 ℃
KIAA1841-R	CATCATGTCCAACTGGAGTCTC	60.1 ℃	55.1 ℃
CDCA2-F	TGCCGAATTACCTCCTAATCCT	60.4 ℃	55.4 ℃
CDCA2-R	TGCTCTACGGTTACTGTGGAAA	61 ℃	56 ℃
PRR11-F	GAAGCTGGCTAACATCATCCTG	60.8 ℃	55.8 ℃
PRR11-R	CTCTGGGTTATGCAGTTCTGG	60.1 ℃	55.1 ℃
TMEM164-F	TCACTTCAGCGTCTTGCAGAT	61.7 ℃	56.7 ℃
TMEM164-R	GCCGTAGAATGGGTCTGAGAT	61.1 ℃	56.1 ℃
GAPDH-F	ACAACTTTGGTATCGTGGAAGG	60.2 ℃	55.2 ℃
GAPDH-R	GCCATCACGCCACAGTTTC	61.7 ℃	56.7 ℃

Table 4 Reaction system of qPCR

component	volume
2 × ChamQ Universal SYBR qPCR Master Mix	10 μL
Forward Primer (10 μM)	0.4 μL
Reverse Primer (10 μM)	0.4 μL
cDNA	2 μL
ddH2O	7.2 μL

Table 5 Reaction condition of qPCR

Step	Step	temperature	time
Pre-denature	Pre-denature	95℃	30 sec
Cycling (40 cycles)	Denature	95℃	10 sec
	Anneal/Extend	54℃	30 sec
Melt curve	Denature	95℃	15 sec
	Anneal	54℃	60 sec
	Denature	95℃	15sec

Table 6 The clinicopathological characteristics of 374 HCC patients in the TCGA cohort

	Overall Cohort Statistics	N
Gender:		374
Female	121 (32.4%)	
Male	253 (67.6%)	
Race list:		374
Unknow	10 (2.67%)	
American Indian or Alaska Native	2 (0.53%)	
Asian	159 (42.5%)	
Black or African American	17 (4.55%)	
White	186 (49.7%)	
Neoadjuvant treatment:		374
No	372 (99.5%)	
Yes	2 (0.53%)	
Status:		374
Alive	283 (75.7%)	
Dead	91 (24.3%)	
Family cancer history:		374
Unknow	51 (13.6%)	
NO	210 (56.1%)	
YES	113 (30.2%)	
Radiation therapy:		374
Unknow	129 (34.5%)	
NO	241 (64.4%)	
YES	4 (1.07%)	

	Overall Cohort Statistics	N
Grade:		374
Unknow	5 (1.34%)	
G1	55 (14.7%)	
G2	179 (47.9%)	
G3	123 (32.9%)	
G4	12 (3.21%)	
Residual tumor:		374
Unknow	7 (1.87%)	
R0	327 (87.4%)	
R1	17 (4.55%)	
R2	1 (0.27%)	
RX	22 (5.88%)	
Child pugh classification grade:		374
Unknow	132 (35.3%)	
A	220 (58.8%)	
B	21 (5.61%)	
C	1 (0.27%)	
Pathologic stage:		374
Unknow	24 (6.42%)	
Stage I	173 (46.3%)	
Stage II	87 (23.3%)	
Stage III	3 (0.80%)	
Stage IIIA	65 (17.4%)	
Stage IIIB	8 (2.14%)	
Stage IIIC	9 (2.41%)	
Stage IV	2 (0.53%)	
Stage IVA	1 (0.27%)	
Stage IVB	2 (0.53%)	

Table 7 The clinicopathological characteristics of 243 HCC patients in the ICGC cohort

	Overall Cohort Statistics	N
	N=243	
Gender:		243
Female	62 (25.5%)	
Male	181 (74.5%)	
Stage:		243
Stage I	37 (15.2%)	
Stage II	109 (44.9%)	
Stage III	76 (31.3%)	
Stage IV	21 (8.64%)	
Prior malignancy:		243
No	213 (87.7%)	
Yes	30 (12.3%)	
Cancer history first degree relative:		243
No	154 (63.4%)	
Unknow	15 (6.17%)	
Yes	74 (30.5%)	
