# Supplementary figures and images for: Development and Validation of the novel Cuproptosis- and Immune-related Signature for Predicting Prognosis in Hepatocellular Carcinoma
Source: J Cancer. 2024 Feb 25;15(8):2260–75. doi: 10.7150/jca.92558 (PMC10937287; doi:10.7150/jca.92558)

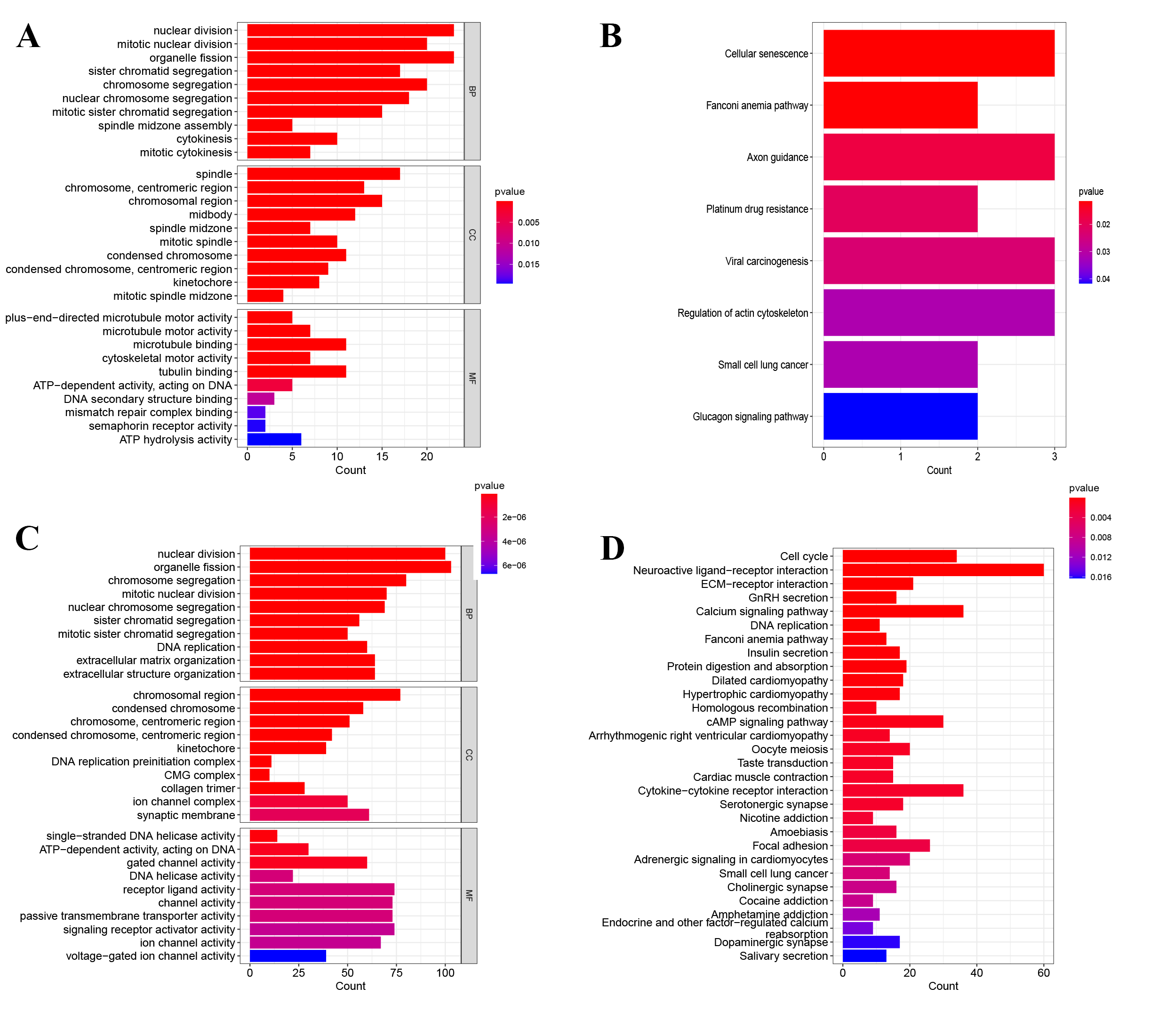

Supplement: Supplementary file 2 — Supplementary figure files. [file jcav15p2260s2.zip › Supplementary Figures/Figure S1.tif]

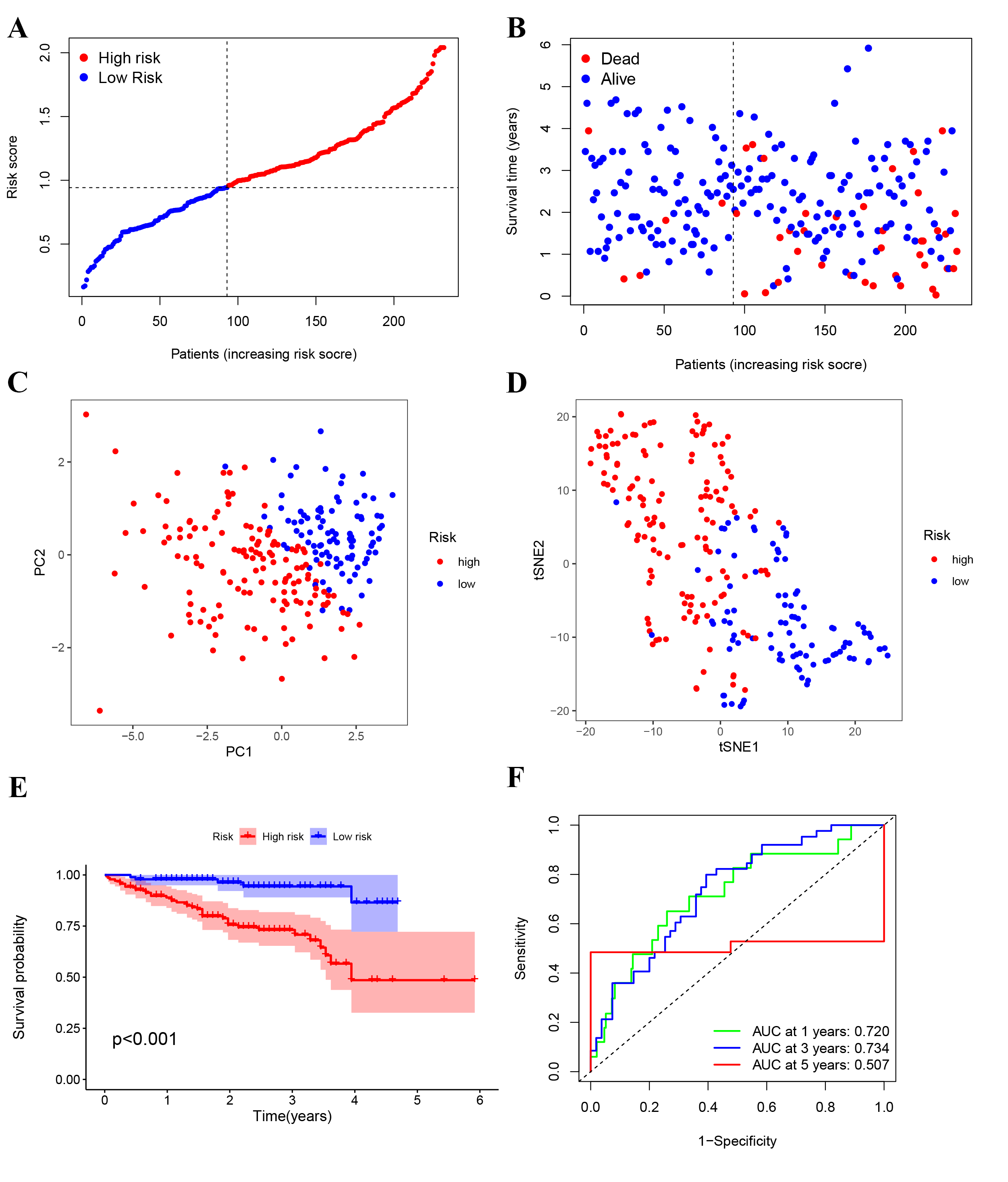

Supplement: Supplementary file 2 — Supplementary figure files. [file jcav15p2260s2.zip › Supplementary Figures/Figure S2.tif]

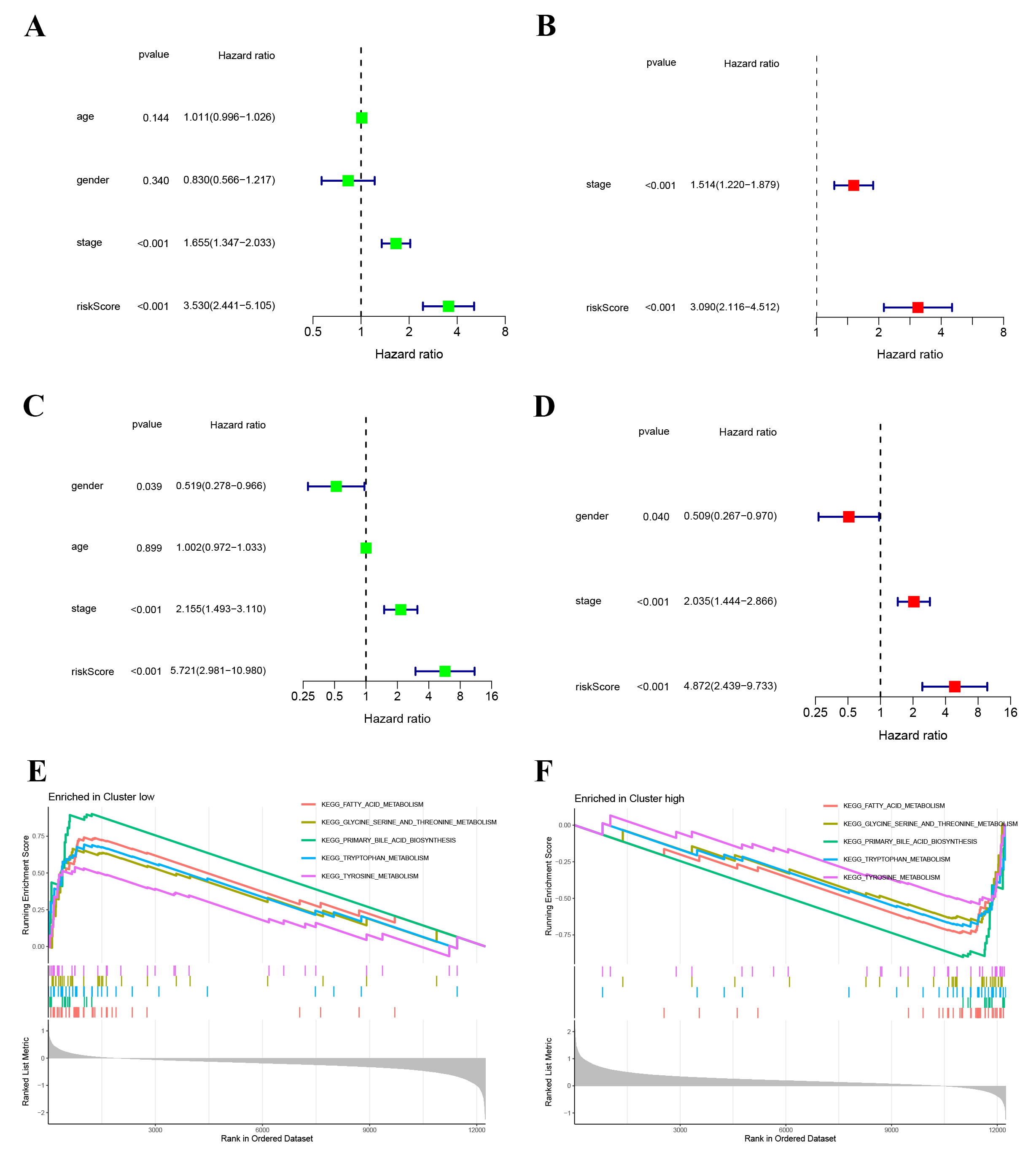

Supplement: Supplementary file 2 — Supplementary figure files. [file jcav15p2260s2.zip › Supplementary Figures/Figure S3.tif]

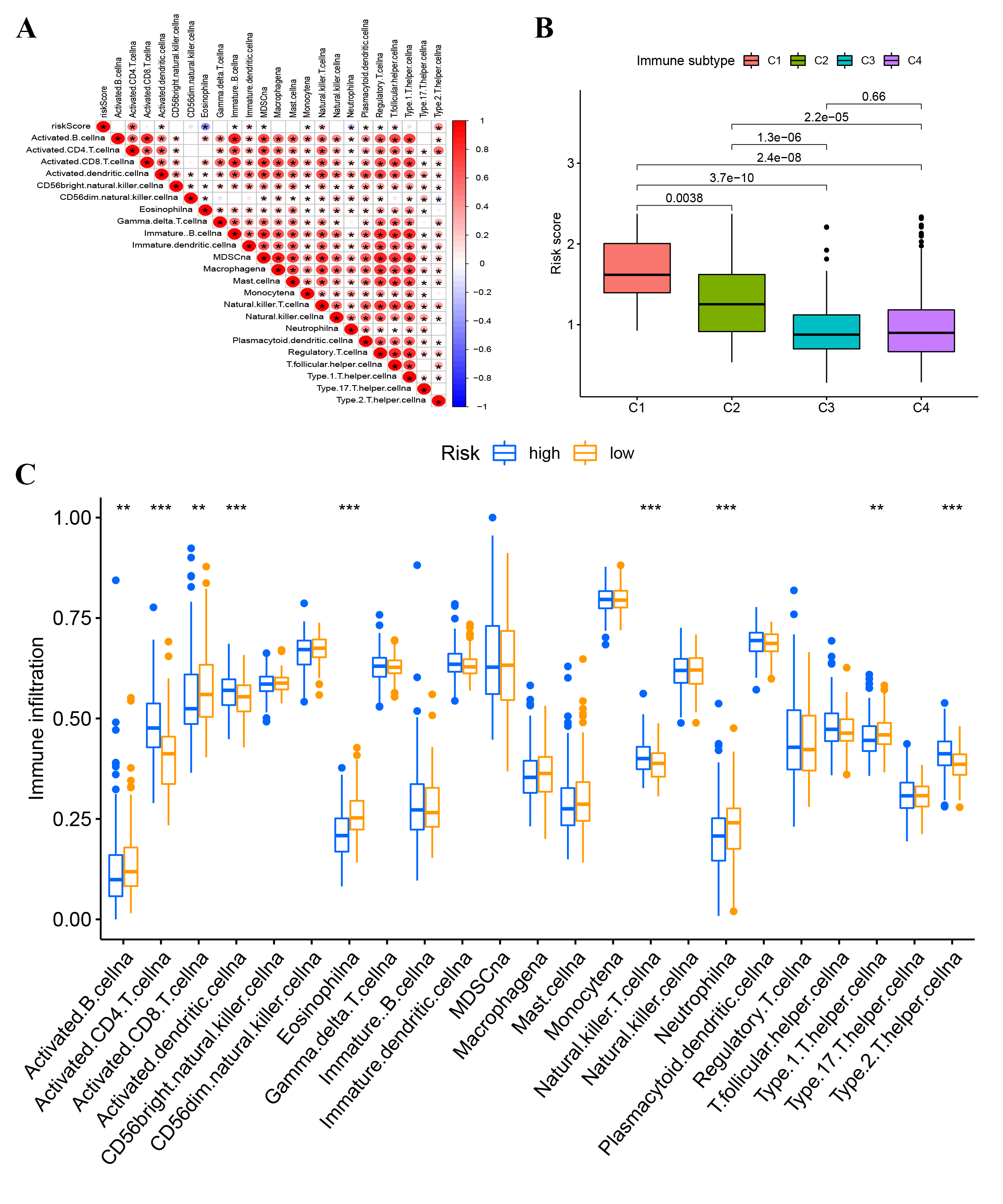

Supplement: Supplementary file 2 — Supplementary figure files. [file jcav15p2260s2.zip › Supplementary Figures/Figure S4.tif]

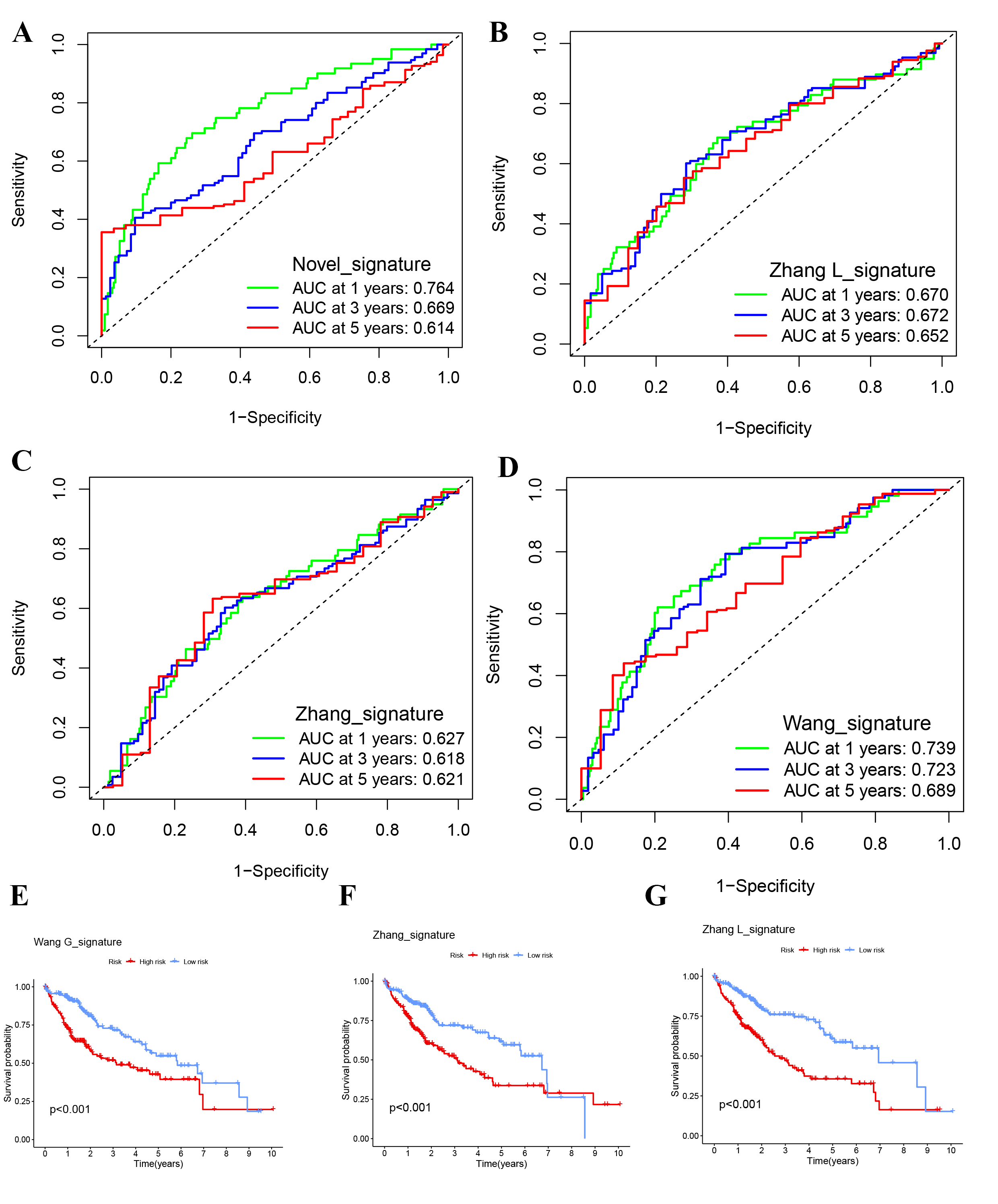

Supplement: Supplementary file 2 — Supplementary figure files. [file jcav15p2260s2.zip › Supplementary Figures/Figure S5.tif]

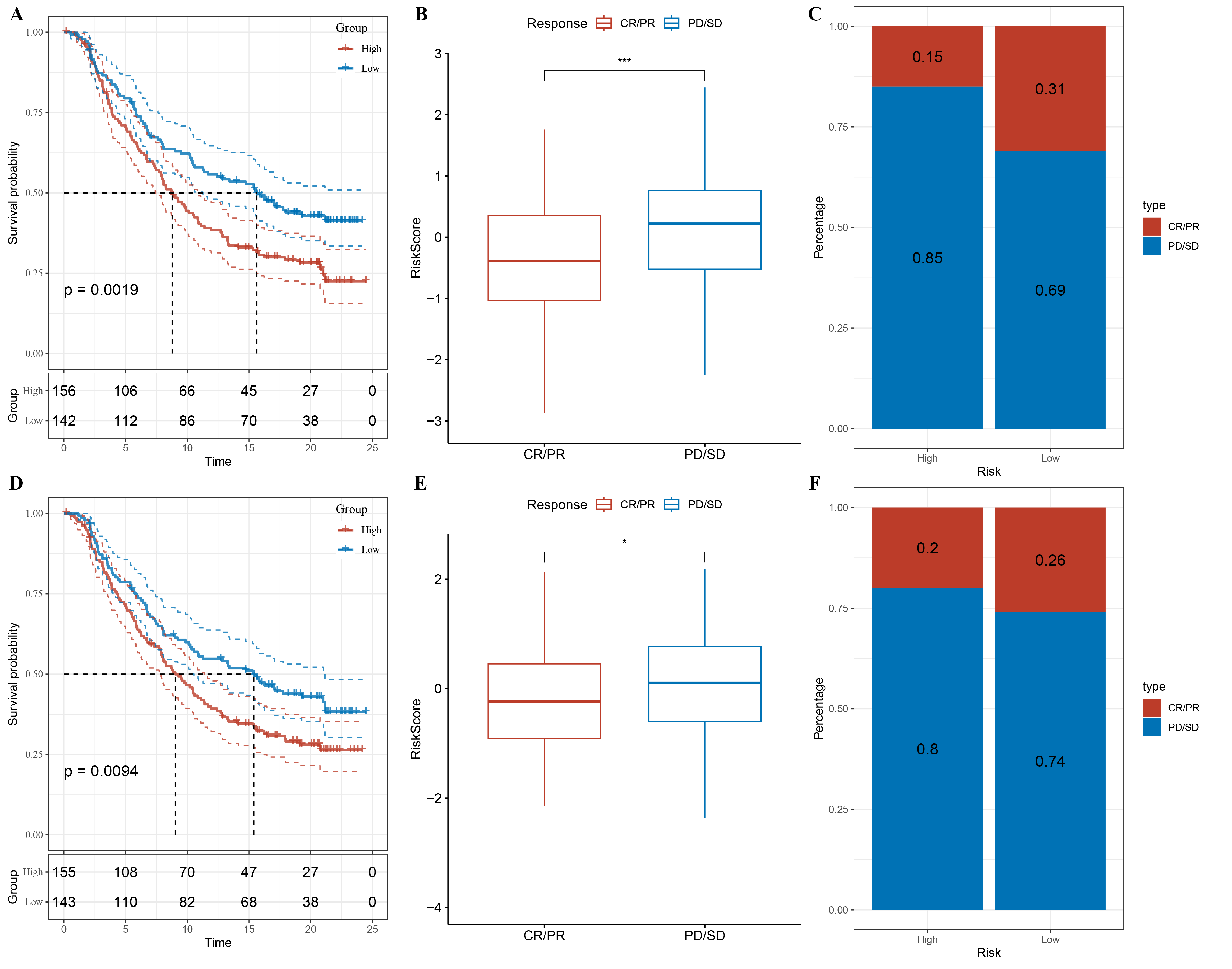

Supplement: Supplementary file 2 — Supplementary figure files. [file jcav15p2260s2.zip › Supplementary Figures/Figure S6.tif]
